# Antioxidant and phytometabolite profiles of ethanolic extract from the cascara pulp of *Coffea arabica *collected from Gayo Highland: A study for potential anti-photoaging agent

**DOI:** 10.12688/f1000research.126762.2

**Published:** 2023-09-18

**Authors:** Wahyu Lestari, Kartini Hasballah, M. Yulianto Listiawan, Sofia Sofia

**Affiliations:** 1 Department of Dermatology, Dr. Zainoel Abidin General Hospital, Banda Aceh, 24415, Indonesia; 2 Department of Dermatology, School of Medicine, Universitas Syiah Kuala, Banda Aceh, 23111, Indonesia; 3 Doctoral Program in Medical Science, School of Medicine, Universitas Syiah Kuala, Banda Aceh, 23111, Indonesia; 4 Department of Pharmacology, School of Medicine, Universitas Syiah Kuala, Banda Aceh, 23111, Indonesia; 5 Department of Dermatology, Faculty of Medicine,, Universitas Airlangga, Surabaya, 60131, Indonesia; 6 Department of Biochemistry, School of Medicine, Universitas Syiah Kuala, Banda Aceh, 23111, Indonesia; 7 Master of Public Health, School of Medicine, Universitas Syiah Kuala, Banda Aceh, 23111, Indonesia

**Keywords:** AP-1, DPPH, flavonoid, in-silico, natural product, polyphenol

## Abstract

**Background**: As the most abundant coffee by-product, cascara pulp has been considered a good source of antioxidants which could be used to prevent photoaging. The aim of this study was to determine the phytometabolite profiles, antioxidant and photoaging properties of the ethanolic extract of *Coffea arabica *cascara pulp.

**Methods**: Ethanolic maceration was performed on the fine powder of *C. arabica *cascara pulp collected from Gayo Highland, Aceh Province, Indonesia. The filtrate obtained was evaluated for its 2,2-diphenyl-1-picrylhydrazyl (DPPH) scavenging activity, total phenolic content (TPC), and total flavonoid content (TFC). The phytometabolite profiling was conducted qualitatively using reagents and quantitatively using gas chromatography—mass spectroscopy (GC-MS). The potential of the cascara pulp phytometabolites in inhibiting activator protein-1 (AP-1) was evaluated through molecular docking.

**Results**: The extract had TPC and TFC of 2.04 mg gallic acid equivalent/g extract and 91.81 mg quercetin equivalent/g extract, respectively. The half-maximal inhibitory concentration (IC _50_) for the DPPH inhibition reached as low as 9.59 mg/L. Qualitative phytocompound screening revealed the presence of alkaloids, saponins, tannins, flavonoids, steroids, quinones, polyphenols, and triterpenoids. GC-MS revealed the extract containing 5-hydroxy-methylfurfural (22.31%); 2,5-dimethyl-4-hydroxy-3(2H)-furanone (0.74%); and caffeine (21.07%), which could form interaction with AP-1 with binding energies of -172.8, -150.8, and -63.188 kJ/mol, respectively.

**Conclusion**: Ethanolic extract from *C. arabica* cascara pulp potentially have anti-photoaging properties which is worthy for further investigations in the future.

## Introduction

Aging, a process that manifests in excessive wrinkle, cutis laxa, degraded collagen and hyperpigmentation, occurs naturally. Other than endogenous factors, the process could be accelerated by exogenous factors. ^1^ Exogenous aging is caused by the surrounding environment that could enhance the oxidative stress in skin tissue including but not limited to cigarette smoke, air pollution, and UV exposure. ^1^^,^^2^ The chronic exposure of solar radiation could also contribute to the development of skin cancer. ^3^ As an addition, maintaining youthful appearance has been the goal of many individual recently which create multibillion-dollar industry of anti-aging products. ^4^

UV radiation from the sun contains three spectra components including UV-A (320-400 nm), UV-B (280-320 nm), and UV-C (100-280 nm). ^5^ UV rays from the sun are known to accelerate the aging process of the skin by inducing oxidative stress that inhibits collagen synthesis. ^6^ Photoaging occurs at the molecular level by UV-induced matrix metalloproteinases (MMPs), which degrade skin collagen. UV stimulates growth factor and cytokine receptors on keratinocytes and fibroblasts, resulting afterward activates mitogen-activated protein (MAP) kinase pathways, leading to downstream signal transduction for the pathophysiology of photoaging in human skin. ^7^ An increase in transcription factors including activator protein-1 (AP-1) and nuclear factor kappa B (NF-κB) is also produced according to the effects of oxidative stress. Gene regulation in response to cell proliferation and differentiation depends on regulation by the transcription factor AP-1. ^8^^–^^10^ Clinical indications of photoaging are based on age, gender, particularly skin, and ethnic and the clinical signs include wrinkles, sagging, roughness, and telangiectasia. ^11^^–^^13^ The main effect that chronic UV radiation on the skin is the reduction of the skin's extracellular matrix by collagen degradation and is considered the main cause of the appearance of wrinkles in photoaged skin. ^5^^–^^14^ Therefore, searching for anti-photoaging has been a crucial research topic in the field of dermatology.

Natural products have been studied for their ability in providing photoprotection, hence preventing photoaging. ^4^^,^^15^ These natural products usually have high antioxidant activities which could suppress the excessive UV radiation-induced reactive oxygen species (ROS) generation. ^16^ *Coffea arabica* and its by-products have been reported multiple times containing abundant antioxidants. ^17^^,^^18^ Cascara pulp is one of the widely studied coffee by-products which have been utilized in food and beverage products. ^19^^–^^21^ Previously, cascara pulp has been extracted using water, methanol, or even supercritical CO _2_. ^22^^,^^23^ However, the use of ethanol solvent that could attract polar compounds and several non-polar compounds is scarcely reported. ^24^ The present study tried to close the gap by determining the phytometabolite profile of the ethanolic extract of *C. arabica* cascara pulp along with its antioxidant properties. Furthermore, the potential of the cascara pulp extract as photoaging agent has been evaluated *in-silico*.

## Methods

### Materials

Materials used in this study included methanol, ethanol 96%, ascorbic acid, gallic acid, quercetin, potassium acetate, and sodium carbonate. Reagents for phytochemical screening included AlCl _3_ powder, Mg powder, Liebermann-Burchard reagent, Folin–Ciocalteu reagent, Mayer reagent, Dragendorf reagent, and Wagner reagent. All materials and reagents were analytical grade and procured from Merck, Selangor, Malaysia. Other reagents were procured from specific companies as described in each section.

### Extraction of cascara pulp

The cascara of *C. arabica* was collected from Gayo Highland, Aceh Province, Indonesia. Cascara pulp was crushed into small pieces to produce simplicia powder and air-dried. The maceration was performed on the dried simplicia powder using ethanol 96% (Merck, Selangor, Malaysia) with ratio of 1:10 for 7 days. Thereafter, the filtrate was collected and dried in a rotary evaporator (45°C) (N-1300VW, EYELA, Tokyo, Japan).

### DPPH inhibition assay

Antioxidant activity of the cascara pulp extract was evaluated based on 2,2-diphenyl-1-picrylhydrazyl (DPPH) assay as described previously ^25^ with some modifications. DPPH was procured from Merck, Selangor, Malaysia. Briefly, 1 mg extract was dissolved up to 10 mL using ethanol 96% to produce 100 mg/L extract solution, which was further dissolved into 2–10 mg/L. DPPH solution was prepared by adding its powder (7.9 mg) into 50 mL methanol (Merck, Selangor, Malaysia). One mL of DPPH 0.4 mM was added into priorly-dissolved extract, homogenized, and incubated at 37°C for 30 minutes. The absorbance was measured at 517 nm on UV-Vis spectrophotometer (Uvmini-1250, Shimadzu, Kyoto, Japan). Ascorbic acid (Merck, Selangor, Malaysia) was used as the control.

### Determination of total phenolic (TPC) and flavonoid contents (TFC)

The total phenolic (TPC) was determined using Folin–Ciocalteu method as described previously. ^26^^,^^27^ Briefly, the cascara pulp extract (0.2 mL) was added into 15.8 mL distilled water which had been pre-added with 1 mL Folin–Ciocalteu reagent (Merck, Selangor, Malaysia). The mixture was shaken rigorously and rested for 8 minutes before added with 15 mL sodium carbonate 10% (Merck, Selangor, Malaysia) and subsequently incubated at room temperature for 2 h. The absorbance was measured at 765 nm using an UV-Vis spectrophotometer (Uvmini-1250, Shimadzu, Kyoto, Japan). The value in concentration was obtained from the calibration curve constructed using gallic acid (gallic acid equivalent (GAE)/g extract) and assigned as total phenolic content (TPC). Gallic acid was purchased from Merck, Selangor, Malaysia.

The total flavonoid content (TFC) was determined using a method as described previously, ^15^ its value was derived from absorbance at 450 nm of a mixture consisting of potassium acetate (0.2 mL) (Merck, Selangor, Malaysia), AlCl _3_ (0.2 mL), and distilled water (5.6 mL) which was incubated for 30 minutes at room temperature (all from Merck, Selangor, Malaysia). TFC was calculated through a calibration curve based on quercetin concentrations (quercetin equivalent (QE)/g extract). Both TPC and TFC measurements followed the procedure reported previously. ^26^

### Qualitative phytochemical screening

Phytochemical screenings followed the procedure reported previously. ^28^^,^^29^ Flavonoids were detected from a reaction between the cascara pulp extract (0.5 in 10 mL methanol) and Mg powder (0.5 mg) in the presence of HCl 0.5 M. As for saponin contents, the extract (0.5) was shaken to form a stable foam after dissolved in 10 mL boiling water. Three reagents, namely Mayer, Dragendorf, and Wagner (all from Merck, Selangor, Malaysia), were used to identify alkaloid contents. Tannins were identified by reacting the extract (0.5 g in 10 mL distilled water) with FeCl _3_ 10%. Quinones was observed through the appearance of red color following the drop-wising of sulfuric acid (1–2 drops) into the extract (0.5 g in 10 mL methanol). As for the steroids and triterpenoids, their presence was indicated by color change following the a few drops addition of reagent Liebermann-Burchard (Merck, Selangor, Malaysia) to the cascara pulp extract (1 g in 10 mL n-hexane).

### Gas chromatography–mass spectroscopy (GC–MS) analysis

The cascara pulp extract was analyzed for its phytometabolites based on gas chromatography–mass spectrometry (GC-MS) (Shimadzu QP2010, Kyoto, Japan). The analysis was carried out with column temperature of 40°C (10°C/minute), flown with He gas. The retention was made 3 minutes (30°C/minute) until the temperature reached 299°C with total running time of 29.63 minutes through splitless injector (280°C; 4.34 psi) with total current of 8.4 mL/m in a 30 m-long Rti-1MS column. Data from mass spectrometer was compared with the database from National Institute of Standards and Technology (NIST).

### Molecular docking

To perform the molecular docking, 3D molecular structure was downloaded from PubChem for the following compounds: caffeine (ID: 2519); 5-hydroxymethylfurfural (ID: 237332); and 2,5-dimethyl-4-hydroxy-3(2H)-furanone (ID: 538757). As for the protein, activator protein-1 (AP-1; ID: 5vpf), its structure was downloaded from RSCB Protein Data Bank. Preparation on the protein and active sites prediction was carried out on Molegro Virtual Docker 5. The grid box position was X: -16.5A; Y: 55.64A; Z: -32.78A. The ligand-protein interaction and its visualization were performed on PyMol and Discovery Studio v.21.1.1, respectively.

## Results

### Antioxidant properties

Antioxidant properties of *C. arabica* cascara pulp were investigated based on the TPC, TFC, and DPPH assay. The TPC and TFC values of the cascara pulp extract were presented in Table 1. The calibration curves obtained from the measurement of gallic acid and quercetin had R ^2^ values of 0.9284 and 0.9868, respectively. When the extract was reacted with Folin–Ciocalteu reagent, the UV-Vis absorbance at 765 nm was found to be 0.055 a.u. Meanwhile, the absorbance of 1.545 a.u. was obtained following the reaction between the reaction with AlCl _3_ and CH _3_COOK. The foregoing absorbance gave the TPC and TFC of the cascara pulp extract as much as 2.04 mg GAE/g extract and 91.81 mg QE/g extract, respectively.

**Table 1.** Antioxidant capacity of the ethanolic extract from the cascara pulp based on total phenolic content and total flavonoid content.

	Phenolic	Flavonoid
Calibration equation	y = 0.0143x – 0.0258	y = 0.0166x + 0.0209
R ^2^	0.9284	0.9868
Absorbance	0.055 a.u.	1.545 a.u.
Total content	2.04 mg GAE/g extract	91.81 mg QE/g extract

GAE: gallic acid equivalent; QE: quercetin equivalent.

**Figure 1.**
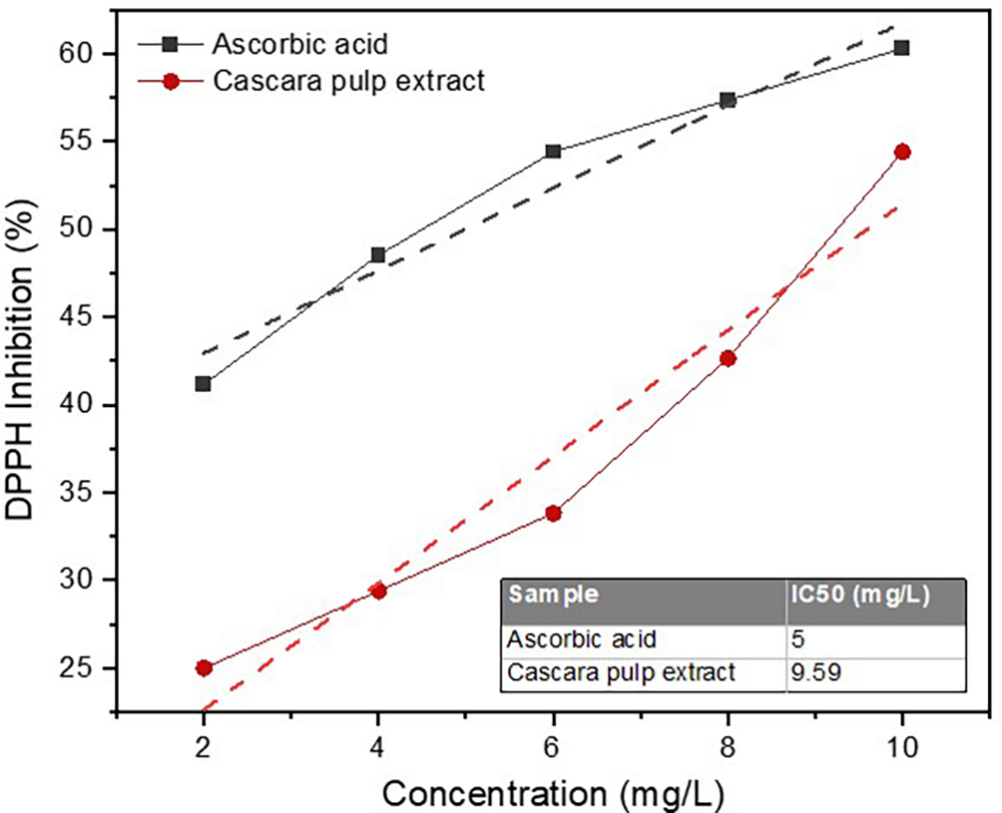
DPPH inhibition by cascaara pulp extract and cascorbid acid (control).

DPPH inhibition percentages yielded by the extract with concentrations ranged from 2 to 10 mg/L have been presented ( Figure 1). When the extract concentrations were at 2, 4, 6, 8, and 10 mg/L, the DPPH inhibition percentages reached 25, 29.41, 33.82, 42.65, and 54.41%, respectively. Based on these values, the linear equation with a slope and intercept of 3.603 and 15.44, respectively (R ^2^ = 0.933). As for the ascorbic acid (control), at the same concentration range, the DPPH inhibitions were 41.18–60.29%. The IC _50_ value of the cascara pulp extract in scavenging free radicals DPPH was 9.59 mg/L, which could be considered highly active as an antioxidant. However, the value still fell short by almost 2-fold when compared with the ascorbic acid (5 mg/L).

### Qualitative phytometabolite screening

Results of the qualitative phytometabolites screening of *C. arabica* cascara pulp have been presented in Table 2. Positive results were indicated by the Dragendorf and Wagner tests, though the phytometabolites were not sensitive to Mayer test. Members of saponin, steroid, quinone, and tannin families were also found positive in the extract. Flavonoids and polyphenols, widely known antioxidant compounds, were shown to be contained in the extract. The presence of triterpenoids was not observable by the qualitative screening.

**Table 2.** Qualitative screening results of the phytometabolites contained in the extract.

Phytometabolite	Screening result
Alkaloids	
Dragendorf	+
Mayer	-
Wagner	+
Saponins	+
Tannins	+
Flavonoids	+
Steroids	+
Quinones	+
Polyphenols	+
Triterpenoids	-

(-) and (+) indicate the presence and absence of the phytometabolites, respectively.

### Phytometabolite profile based on GC–MS

**Figure 2.**
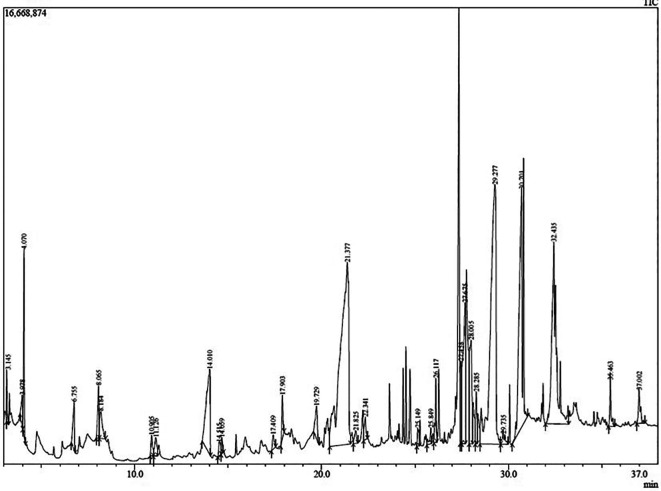
Chromatogram of cascara pulp extract.

After the initial screening, GC–MS analysis was carried out to identify the phytoconstituents in the extract. The chromatogram depicting peaks from each phytometabolite detected has been presented in Figure 2. Details of the recorded compounds along with the quantitative information (peak area which could be associated to the metabolite contents) have been presented in Table 3. As many as 30 phytocompounds were identified in this present study. Compounds 5-hydroximethylfurfural; caffeine; n-hexadecanoic acid; cis-vaccenic acid; and 2(1H)-naphthalenone, 3,5,6,7,8,8a-hexahydro-4, 8a-dimethyl-6-(1-methylethenyl) topped the phytometabolite abundance rank in the extract with peak areas of 22.31, 21.07, 12.85, 11.94, and 5.26%, respectively. However, only 5-hydroxy-methylfurfural; 2,5-dimethyl-4-hydroxy 3(2H)-furanone; and caffeine (peak area = 22.31, 0.74, and 21.07%, respectively) were considered most bioactive based on previous research. ^30^^–^^32^ The aforementioned phytocompounds were then selected for molecular docking study against AP-1.

**Table 3.** Phytometabolites of the ethanolic extract as identified by the GC–MS.

Retention time (min)	Phytometabolite	Peak area (%)
3.145	Formic acid	0.54
3.978	Acetic acid (CAS) ethylic acid	0.60
4.070	Propanal, 2-oxo- (CAS) pyruvaldehyde	2.08
6.755	Propanoic acid, 2-oxo-, methyl ester (CAS) methyl pyruvate	1.04
8.065	Ethane, 1,1′,1″-[methylidynetris (oxy)]tris-(CAS) triethyl formate	1.03
8.184	2-Furancarboxaldehyde (CAS) furfural	1.07
10.905	2-Propanone, 1-(acetyloxy)-	0.47
11.126	2-Furanmethanol	0.66
14.010	Isopropenylacetic acid	4.85
14.515	5-Methyl furfural	0.41
14.659	2-Furancarboxaldehyde, 5-methyl-(CAS) 5-methyl-2-furfural	0.28
17.409	Orcinol	0.37
17.903	2,5-Dimethyl-4-hydroxy-3(2H)-furanone	0.74
19.729	2,3-Dihydro-3,5-dihydroxy-6-methyl-4H-pyran-4-one	1.03
21.377	5-Hydroximethylfurfural	22.31
21.825	5-Acetoxymethyl-2-furaldehyde	0.38
22.341	2-Propoxy-succinic acid, dimethyl ester	0.59
25.149	Phenol, 3,5-bis(1,1-dimethylethyl)-	0.40
25.849	9-Octadecenoic acid (Z)- (CAS) oleic acid	0.37
26.117	1H-Cycloprop [e]vazulen-4-ol, decahydro-1,1	0.93
27.458	2,6,10,14-Hexadecatetraen-1-ol, 3,7,11,15-tetramethyl, acetate (E,E,E)	1.52
27.675	2(1H) Naphthalenone, 3,5,6,7,8,8a-hexahydro-4, 8a-dimethyl-6-(1-methylethenyl)	5.26
28.005	Hydrazinecarboxamide, 2-(2-methylcyclohexylidene)	3.93
28.285	Tetradecanoic acid	0.92
29.277	Caffeine	21.07
29.735	Corymbolone	0.51
30.701	n-Hexadecanoic acid	12.85
32.435	Cis-Vaccenic acid	11.94
35.463	Hexadecanoic acid, 2-hydroxy-1-(hydroxym	0.91
37.002	9-Octadecenoic acid (Z)-, 2,3-dihydroxypropyl ester (CAS) 1-monoolein	0.95

### *In-silico* interactions between the identified phytometabolites and AP-1

**Figure 3.**
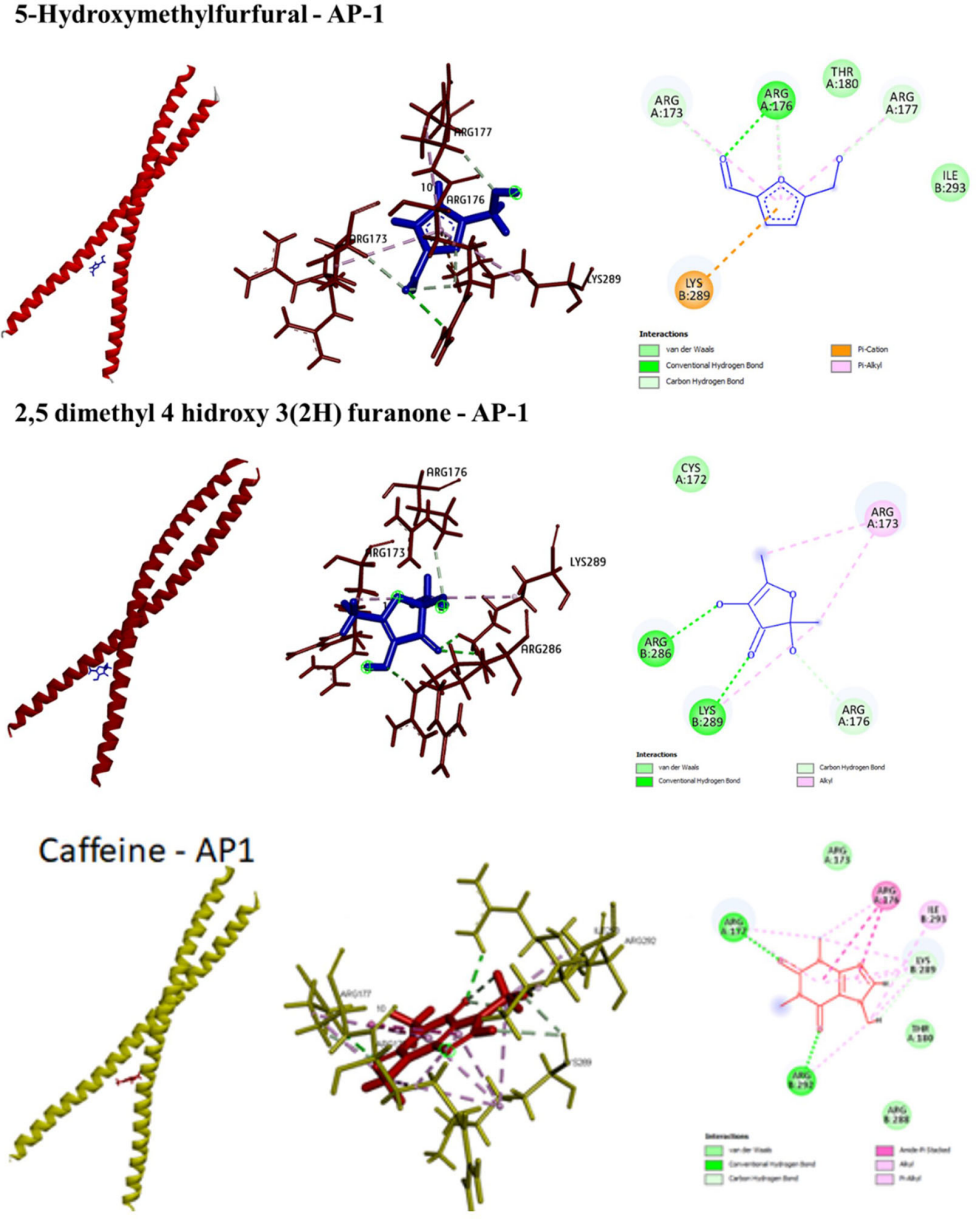
3D (left and middle) and 2D (right) conformations of the interactions between the phytometabolites and AP-1.

The summaries of molecular docking results were presented in Table 4. Highest binding energy was exhibited by 5-hydroxy-methylfurfural (-172.8 kJ/mol) attributed to six hydrogen bonds and five Van der Waals interactions. The second most potential AP-1 inhibitor was 2,5-dimethyl-4-hydroxy-3(2H)-furanone with a binding energy of -150.8 kJ/mol involving four H bonds and three non-polar interactions. Caffeine appeared to have the lowest affinity with AP-1, where the binding energy only reached -63.188 kJ/mol despite the seven H bond, six alkyl, and four pi-alkyl interactions. All the three compounds formed interactions with AP-1 through ARG176, ARG177, and LYS289. While both 5-hydroxy-methylfurfural and 2,5-dimethyl-4-hydroxy-3(2H)-furanone interacted with ARG173, only caffeine that did not. The 3D representations of the foregoing interactions have been presented in Figure 3.

**Table 4.** Molecular docking results of the interaction between the phytometabolites and AP-1.

Ligand	Binding energy (kJ/mol)	Interaction	Distance (Å)	Interaction types
5-Hydroxy-methylfurfural	-172.8	A:ARG176:HH11 - :10:O3	2.64	Conventional H bond
		A:ARG173:HA - :10:O3	2.42	Carbon H bond
		A:ARG173:HA:B - :10:O3	2.44	Carbon H bond
		A:ARG176:HD1 - :10:O1	2.64	Carbon H bond
		A:ARG176:HD1 - :10:O3	2.47	Carbon H bond
		A:ARG177:HA - :10:O2	2.45	Carbon H bond
		B:LYS289:NZ - :10	4.25	Pi-cation
		:10 - A:ARG173	5.13	Pi-alkyl
		:10 - A:ARG176	3.71	Pi-alkyl
		:10 - A:ARG177	4.91	Pi-alkyl
		:10 - B:LYS289	4.62	Pi-alkyl
2,5-Dimethyl-4-hydroxy-3(2H)-furanone	-150.8	B:ARG286:HE - :10:O3	2.23	Conventional H bond
		B:LYS289:HZ1 - :10:O4	2.54	Conventional H bond
		B:LYS289:HZ2 - :10:O4	2.39	Conventional H bond
		A:ARG176:HD1 - :10:O2	2.63	Carbon H bond
		:10:C5 - A:ARG173	4.15	Alkyl
		:10:C5 - B:LYS289	4.67	Alkyl
		:10:C6 - A:ARG173	4.35	Alkyl
Caffeine	-63.188	A:ARG177:HH11 - :10:O2	2.24021	Conventional H bond
		B:ARG292:HH11 - :10:O1	2.37319	Conventional H bond
		A:ARG177:HD2 - :10:O2	2.61266	Carbon H bond
		:10:H1 - B:LYS289:O	2.98414	Carbon H bond
		:10:H5 - B:LYS289:O	2.40609	Carbon H bond
		:10:H6 - :10:O1	2.83378	Carbon H bond
		:10:H7 - :10:O1	2.83398	Carbon H bond
		A:ARG176:C,O;ARG177:N - :10	5.3792	Amide-pi stacked
		A:ARG176:C,O;ARG177:N - :10	4.6741	Amide-pi stacked
		:10:C6 - A:ARG176	4.19428	Alkyl
		:10:C6 - A:ARG177	3.96796	Alkyl
		:10:C6 - B:LYS289	4.9672	Alkyl
		:10:C7 - B:LYS289	4.63265	Alkyl
		:10:C7 - B:ARG292	3.32205	Alkyl
		:10:C7 - B:ILE293	5.2454	Alkyl
		:10 - A:ARG176	4.67272	Pi-Alkyl
		:10 - A:ARG177	5.06042	Pi-Alkyl
		:10 - B:LYS289	4.24254	Pi-Alkyl
		:10 - B:LYS289	3.70068	Pi-Alkyl

## Discussion

In this present study, the antioxidant activity of *C. arabica* cascara pulp could be considered high since the IC _50_ value of DPPH inhibition reached below 100 mg/L. ^26^ The antioxidant properties of the cascara pulp extract are further supported by the TPC and TFC of 2.04 mg GAE/g extract and 91.81 mg QE/g extract, respectively. *C. arabica* has previously been reported to possess high antioxidant activities. A study in India revealed that 200 mg/L *C. arabica* seed extract could yield >90% DPPH inhibition. ^18^ The authors attributed the finding with the donation of hydrogen from hydroxyl groups of the bioactive metabolites which could stop the oxidation process by DPPH via stable end products formation. ^18^ Other studies also observed the strong antioxidant activity of *C. arabica* along with its high TPC. ^33^^,^^34^ In our previous systematic review, we found there are at least 13 studies confirming the antioxidant activities of *C. arabica* by-products. ^17^ IC _50_ value obtained from the DPPH assays of arabica coffee husk using supercritical fluid extraction was >0.25 mg/L. ^23^ Arabica coffee pulp extracted with distilled water was reported to contained TPC as high as 0.28 mg GAE/g extract. ^35^ Hence, our ethanolic extract from *C. arabica* cascara pulp had higher DPPH scavenging activity and TPC value than that of previously studied by-products.

UV radiation could induce aging process (photoaging) by promoting reactive oxygen species (ROS) production which could result in excessive oxidative stress and inflammation in skin. Indeed, ROS is naturally produced in mitochondria resulted from the aerobic metabolism, particularly in electron transport chain, which contributes to slow endogenous aging. ^36^ However, there are studies who suggest that ROS production could be enhanced by UV light exposure. Activities of keratinocytes and fibroblasts in producing ROS are positively correlated with the UV radiation intensity. ^37^^,^^38^ The increase of ROS release following the solar light exposure could also attributed to the downregulation of ROS neutralizing enzymes *viz* glutathione reductase and peroxidase. ^1^ In addition, ROS could mediate several signaling pathways to induce skin damage. ^16^^,^^37^^,^^39^ A terpenoid isolated from an ethyl acetate fraction of coffee silverskin was reported to be capable of inhibiting ROS production and potential as photoaging agent. ^22^ Taken altogether, the cascara pulp obtained herein is potential in preventing photoaging attributed to its high antioxidant activity that could suppress the oxidative stress in skin tissue following solar radiation.

ROS induced from the UV exposure could initiate the cascading reaction of mitogen-activated protein kinases (MAPKs), consisting of extracellular C-Jun N-terminal kinase (JNKs), signal-regulated kinase (ERK), and p38MAPK. ^40^ The activation of JNK and p38MAPK pathways promotes activator protein-1 (AP-1) and cyclooxygenase-2 (COX-2). ^40^ This reaction cascade contributes to the enhanced levels of interleukin (IL)-8, IL-10, prostaglandin G2, and vascular endothelial factor which are responsible for inflammatory reaction, differentiation, immunosuppression, proliferation, as well as angiogenesis. ^1^ Moreover, AP-1 is responsible to aging process by both inhibiting collagen synthesis via direct binding with procollagen promoter and degrading collagen via interaction with matrix metalloproteinase 1 (MMP1). ^41^^,^^42^ Based on the foregoing explanations, researchers have used AP-1 as the target for anti-photoaging agents. ^43^^–^^45^ Hence, we had performed molecular docking studies to observe the potential of the phytometabolites from the cascara pulp extract to interact with AP-1.

In this present study, two predominant compounds (5-hydroxy-methylfurfural and caffeine) and one minor compound (2,5-dimethyl-4-hydroxy-3(2H)-furanone) were shown to have strong affinity with AP-1 through molecular docking studies. From the highest to the lowest, the phytometabolites could be ranked as follows: 5-hydroxy-methylfurfural > 2,5-dimethyl-4-hydroxy-3(2H)-furanone > caffeine. Both 5-hydroxy-methylfurfural and 2,5-dimethyl-4-hydroxy-3(2H)-furanone are common food additives, while caffeine is a common compound in coffee products. Similarly, a study found that caffeine was among the isolates obtained from antioxidant assay-guided procedure on Robusta coffee seeds, yet its bioactivity is relatively minimum. ^46^ In our previous *in-silico* screening using gallic acid, malonic acid, gallic acid, and decanoic acid from water:ethanol combination extract of *C. arabia* cascara pulp, ARG173, ARG176, ARG177, and LYS289 were also found as common binding sites. ^47^ The highest binding energy found in that previous study was -242.24 kJ/mol. ^47^ Though the highest binding energy in this present study is -172.8 kJ/mol, obtained from 5-hydroxy-methylfurfural, the value is relatively high and could be considered as an effective binding. ^48^^,^^49^ Moreover, the antioxidant properties of the cascara pulp herein could contribute to other preventive mechanisms of photoaging. Nonetheless, it is worth noting that *in-silico* study alone is not sufficient to conclude the AP-1 inhibition by the cascara pulp extract, where *in vitro* study is a must. ^24^

## Conclusions

The ethanolic extract from the cascara pulp of *C. arabica* is highly active as an antioxidant agent. Phytocompound profiling evidences the antioxidant metabolite richness of the cascara pulp extract which could act as anti-photoaging agent. *In-silico* studies reveal the potential of strong interaction between the bioactive phytometabolites with AP-1. Further research with *in vitro* or *in vivo* design is warranted to confirm the anti-photoaging activities of the phytometabolites contained in the *C. arabica* cascara.

## Data Availability

PubChem: Molecular structure for caffeine. 2519, https://identifiers.org/pubchem.compound:2519. ^50^ PubChem: Molecular structure for 5-hydroxymethylfurfural. 237332, https://identifiers.org/pubchem.compound:237332. ^51^ PubChem: Molecular structure for 2,5-dimethyl-4-hydroxy-3(2H)-furanone. 538757, https://identifiers.org/pubchem.compound:538757. ^52^ PubChem: Molecular structure for activator protein-1. https://doi.org/10.2210/pdb5VPF/pdb. ^53^ Figshare: ‘Antioxidant and phytometabolite profiles of ethanolic extract from the cascara pulp of Coffea arabica collected from Gayo Highland: A study for potential photoaging agent’, https://doi.org/10.6084/m9.figshare.21219323. ^54^ This project contains the following underlying data:
• DPPH Inhibition Assay.docx • Determination of Total Phenolic Content.docx • Determination of Total Flavonoid Content.docx • Calibration Curve_Total flavonoid.png • Calibration Curve_Total Phenolic.png • DPPH inhibition by cascaara pulp extract and cascorbid acid (control).jpg •
Figure 2. Chromatogram of cascara pulp extract.jpg • 3D (left and middle) and 2D (right) conformations of the interactions between the phytometabolites and AP-1.jpg Data are available under the terms of the Creative Commons Attribution 4.0 International license (CC-BY 4.0).
