# Supplementary material for: Antioxidant and phytometabolite profiles of ethanolic extract from the cascara pulp of Coffea arabica collected from Gayo Highland: A study for potential anti-photoaging agent
Source: F1000Res. 2023 Sep 18;12:12. Originally published 2023 Jan 5. [Version 2] doi: 10.12688/f1000research.126762.2 (PMC10523095; doi:10.12688/f1000research.126762.2)
Supplement: Peer review history

# Reviewer report: Reviewer response for version 2

**DOI:** 10.5256/f1000research.152714.r207137

**Published:** 2023-09-26

**Authors:** Maria Atanassova

**Affiliations:** 1 Nutritional Scientific Consulting, Chemical Engineering, University of Chemical Technology and Metalurgy, Sofia, Bulgaria

I have read the manuscript carefully and agree with the corrections the authors have made following my recommendations. I think your manuscript can be accepted for indexing.

Is the work clearly and accurately presented and does it cite the current literature?

Partly

If applicable, is the statistical analysis and its interpretation appropriate?

I cannot comment. A qualified statistician is required.

Are all the source data underlying the results available to ensure full reproducibility?

Yes

Is the study design appropriate and is the work technically sound?

Yes

Are the conclusions drawn adequately supported by the results?

Yes

Are sufficient details of methods and analysis provided to allow replication by others?

Yes

Reviewer Expertise:

My research area is Total Phenols and Total Flavonoids, Bioactive Compounds and Antioxidant Activity of Extracts from Different Natural Plants

I confirm that I have read this submission and believe that I have an appropriate level of expertise to confirm that it is of an acceptable scientific standard.

# Reviewer report: Reviewer response for version 1

**DOI:** 10.5256/f1000research.139202.r171576

**Published:** 2023-05-09

**Authors:** Maria Atanassova

**Affiliations:** 1 Nutritional Scientific Consulting, Chemical Engineering, University of Chemical Technology and Metalurgy, Sofia, Bulgaria

The manuscript "Antioxidant and phytometabolite profiles of ethanolic extract from the cascara pulp of Coffea arabica collected from Gayo Highland: A study for potential photoaging agent" with authors Wahyu Lest, Kartini Hasballah, M. Yulianto Listiawan, Sofia Sofia is interesting and original. I think it would be of interest not only to readers of F1000Research but also to other researchers. I think that the task that the authors have set themselves is very serious.

 In the Introduction part, the authors have cited 10 sources from the last 3-5 years, out of a total of 14. This shows that the authors have worked very seriously on their goal to prove the seriousness of the research they have done. I would like to say that I think the Introduction is too short and I ask the authors to add more sources.

 As for the Methods and Materials section, the methods are generally known, well described, and could be replicated by any researcher. It struck me that two of the methods have no sources noted. I ask the authors to cite where they got the methods because according to the journal's requirements, this omission amounts to plagiarism. These are the methods: DPPH inhibition assay; Determination of total phenolic (TPC). The authors may have modified them in their use but they should also note this.

 Results are presented accurately and clearly. They are supported by relevant tables and figures. Accordingly, the results obtained by the authors enable any reader of this manuscript to get a clear idea. In my opinion, the authors should note Total Phenolics and Total Flavonoids in the table. The phenols and flavonoids for which the authors present the results are not one specific compound and for that they use the corresponding equivalent. For example, if the authors had used another equivalent such as catechin or rutin to prove the flavonoids, I think they would have obtained different results. Another thing that I would like the authors to pay attention to is the formula of potassium acetate CH _3_CO _2_K, in my opinion, it should be written according to IUPAC CH _3_COOK. I also ask the authors to write the formulas of the compounds in the text according to the IUPAC requirements, for example: 2,5-Dimethyl-4-hydroxy-3(2H)-furanone.

 In the Discussion section, I think the authors presented their results accurately and clearly and supported them with relevant citations. In my opinion, the conclusion supports the results obtained by the authors.

Is the work clearly and accurately presented and does it cite the current literature?

Partly

If applicable, is the statistical analysis and its interpretation appropriate?

I cannot comment. A qualified statistician is required.

Are all the source data underlying the results available to ensure full reproducibility?

Yes

Is the study design appropriate and is the work technically sound?

Yes

Are the conclusions drawn adequately supported by the results?

Yes

Are sufficient details of methods and analysis provided to allow replication by others?

Yes

Reviewer Expertise:

My research area is Total Phenols and Total Flavonoids, Bioactive Compounds and Antioxidant Activity of Extracts from Different Natural Plants

I confirm that I have read this submission and believe that I have an appropriate level of expertise to confirm that it is of an acceptable scientific standard, however I have significant reservations, as outlined above.

# Response

**Published:** 2023-06-13

**Authors:** Kartini Hasballah

**Affiliations:** Faculty of Medicine, Syiah Kuala University, Indonesia

We thank and appreciate the reviewer comment regarding our manuscript and we are humbled that our efforts have been well conveyed. We thank and appreciate the reviewer comment about our intention regarding the sources of our manuscript. We’d like to agree about the introduction part and would like to add more sources as soon as possible. Again, we’d like to thank the reviewer regarding her veracity as well as her precision. We apologize about our lack of source, we’d like to correct it soon enough. We’d like to thank the reviewer about the comment regarding our result and we’re content that our purpose have been well received. We’d also appreciate the advice regarding the phenolic and flavonoid matter. About the formula, yes, we’re completely agree that we should write it according to the IUPAC requirement. We’d like to correct it later. We thank and appreciate the reviewer comment regarding the discussion and conclusion section. We really do appreciate the reviewer for taking the time and for giving us her insights in order to make our manuscript better.

# Response

**Published:** 2023-07-06

**Authors:** Kartini Hasballah

**Affiliations:** Universitas Syiah Kuala, Indonesia

Dear Reviewer,

 Thank you for reviewing our article and proving the comments and suggestions. Based on your comments and suggestions we have revised our article:

1. We have added more information about photoaging in Introduction section with nine additional references.
2. We have added the references for both methods of DPPH inhibition assay and total phenolic (TPC).
3. The total of the total phenolics and total flavonoids have been provided in the Table 1.
4. We have revised all the names of the compounds according to IUPAC. We would like to thank you for pick some of them.

Best regards,

 Authors

# Reviewer report: Reviewer response for version 1

**DOI:** 10.5256/f1000research.139202.r165756

**Published:** 2023-03-23

**Authors:** Emil Salim

**Affiliations:** 1 Department of Pharmacology, Faculty of Pharmacy, Universitas Sumatera Utara, Medan, North Sumatra, Indonesia

The authors studied the antioxidant properties of ethanolic extract from the cascara pulp of *Coffea arabica *collected from Gayo Highland. Using the DPPH method, they found that the extract has strong antioxidant activity. Moreover, they studied the phytochemical constituent of the extract using phytochemical screening methods using reagents and found that the extract contained saponin, steroid, quinone, tannin, flavonoids and polyphenols. Using GCMC, they found some bioactive compounds exist in the extract, such as 5-hydroxy-methylfurfural, 2,5 dimethyl 4 hydroxy 3(2H) furanone and Caffeine. By performing in silico study, they showed that these compounds have interaction with AP-1, a target for anti-photoaging agents.

 The study's background, methods, results, discussion, and conclusion have been presented clearly. However, in the title, I would like to suggest that it should be "anti-photoaging agent" rather than "photoaging agent".

 I spotted a tiny error in the sentence: The second most potential AP-1 inhibitor was and 2,5 dimethyl 4 hidroxy 3(2H) furanone with a binding energy of -150.8 kJ/mol involving four H bonds and three non-polar interactions. I think the word "and" should be deleted. Also in some sentences, "hidroxy" should be written as "hydroxy".

Is the work clearly and accurately presented and does it cite the current literature?

Yes

If applicable, is the statistical analysis and its interpretation appropriate?

Not applicable

Are all the source data underlying the results available to ensure full reproducibility?

Yes

Is the study design appropriate and is the work technically sound?

Yes

Are the conclusions drawn adequately supported by the results?

Yes

Are sufficient details of methods and analysis provided to allow replication by others?

Yes

Reviewer Expertise:

Pharmacology and Immunology

I confirm that I have read this submission and believe that I have an appropriate level of expertise to confirm that it is of an acceptable scientific standard.

# Response

**Published:** 2023-07-05

**Authors:** Kartini Hasballah

**Affiliations:** Universitas Syiah Kuala, Indonesia

Dear Reviewer,

 We would like thank you for your suggestions and corrections. We have revised our title from "photoaging agent" to "anti-photoaging agent". We also have replaced all "hidroxy" to "hydroxy" in the entire article.

 Authors
